# Evaluation of the comparative efficacy and safety of surgical strategies for long bone defects: a network meta-analysis

**DOI:** 10.1097/JS9.0000000000002283

**Published:** 2025-01-29

**Authors:** Yuanli Shen, Qihong Yang, Hui Cheng, Yitian Feng, Yuan Liu, Jun Hu

**Affiliations:** a Department of Infectious Diseases, the First Affiliated Hospital of Nanjing Medical University, Nanjing, China; b Department of Orthopedics, the First Affiliated Hospital of Nanjing Medical University, Nanjing, China

**Keywords:** bone defects, bone regeneration, complications, meta-analysis, surgery

## Abstract

**Background:**

To evaluate the safety and efficacy of various surgical treatments for long bone defects. Despite numerous observational studies, randomized controlled trials (RCTs), and meta-analyses, the optimal surgical treatment for long bone defects remains undetermined.

**Methods:**

A network meta-analysis (NMA) was conducted. PubMed, Embase, and the Cochrane Library were searched for articles published between 1 January 2000 and 12 January 2023 on surgical treatments for long bone defects. RCTs and observational studies comparing five surgical treatments were selected: the Masquelet technique (MT), bone transport (BT), vascularized bone graft (VBG), non-VBG (NVBG), and bone tissue engineering (BTE). Data were extracted by two independent reviewers. The NMA aggregated direct and indirect evidence. Treatments were ranked using the surface under the cumulative ranking curve (SUCRA) scores. Data are presented as mean differences and 95% confidence intervals. The primary outcomes were the postoperative healing rate, with subgroup analysis based on defect size (4–8 cm and >8 cm). The secondary outcomes included postoperative complications.

**Results:**

This NMA included 23 studies (three RCTs and 20 observational studies) with 930 participants (median age, 35 years). There were no significant differences in clinical outcomes among the treatments. VBG (SUCRA, 75.1%) was rated as optimal for healing, and BTE (SUCRA, 28.5%) was the least effective. BTE had the highest complication rate (SUCRA, 90.9%), whereas NVBG had the lowest complication rate (SUCRA, 27.6%). Subgroup analysis showed reduced heterogeneity: for 4–8 cm defects, VBG (SUCRA, 80.4%) was optimal, and for >8 cm defects, BT (SUCRA, 76.2%) was optimal.

**Conclusions:**

VBG and BT may offer superior clinical outcomes for long bone defects compared to MT, NVBG, and BTE. However, BTE is associated with a high risk of complications. Further high-quality, large-sample RCTs are required to confirm these findings.

## Introduction

Globally, there are more than 20 million patients with bone defects^[1]^. Only in Europe and America, more than half a million patients require bone-defect repair treatments annually, with total expenses surpassing $3 billion and an average treatment cost of $5000 per patient^[2,3]^. In addition to the economic burden, complications associated with bone defects are severe. Numerous researchers believe that when a bone defect exceeds the critical size threshold (approximately >2 cm) or when the bone circumference is >50%, it can lead to nonunion, malunion, or pathological fractures, among other serious complications^[4,5]^. Reports indicate that approximately 5–10% of fractures exhibit delayed healing or nonunion, whereas nearly all segmental bone defects result in fracture nonunion^[1,6]^. Hence, it is crucial to perform early surgical interventions for patients with bone defects to save limb function, ensure physical and mental health, and alleviate the economic burden.

**Highlights**

- This study applied a network meta-analysis method to analyze data from 3 randomized controlled trials and 20 observational studies, comparing the effectiveness and safety of different surgical methods for long bone defects, which has not been reported in previous studies.
- Network meta-analysis of surgical treatments for long bone defects showed VBG and BT may provide superior outcomes compared to MT, NVBG, and BTE, which had the highest complication rate. Further large-sample RCTs are needed to confirm these findings.
- The findings of this study provide healthcare professionals and patients with valuable tools for improving clinical decision-making, and are expected to enhance the overall quality and efficiency of healthcare services.

Currently, surgical interventions for long bone defects include bone grafting, vascularized fibular grafting (VBG), bone transport (BT), the Masquelet technique (MT), and bone tissue engineering (BTE), among others^[7–10]^. However, there is significant controversy regarding the best treatment option for long bone defects despite numerous meta-analyses comparing these options. Kai *et al* believe that for treating bone defects caused by infection, MT has a higher rate of bone-related complications, whereas BT offers better functional outcomes^[11]^. On the other hand, Ren *et al* argue that MT, compared to BT, has advantages in treating lower limb infectious bone defects, such as shorter healing time, lower complication rates, and better postoperative quality of life^[12]^. However, Wakefield *et al* found no clinical differences in outcomes between BT and MT in patients with segmental bone defects^[13]^.

Traditional meta-analyses are limited in determining the optimal treatment for long bone defects because they only allow for pairwise comparisons, which hinders a comprehensive evaluation of multiple interventions simultaneously. This limitation underscores the need for a more advanced analytical method. Network meta-analysis (NMA) addresses this challenge by synthesizing both direct and indirect evidence, enabling the simultaneous quantitative comparison of multiple treatments within a single framework. NMA not only assesses the relative efficacy and safety of various interventions but also ranks them according to their effectiveness. This provides clinicians with a clear, evidence-based hierarchy of treatment options, which is essential for informed clinical decision-making in complex cases involving multiple treatment strategies^[14–16]^.

Therefore, this study conducted an NMA to compare the healing and postoperative complication rates of different surgical treatment methods in order to systematically evaluate the safety and effectiveness of surgical treatments for bone defects.

## Methods

### Protocol and registration

We organized a team of experts, including orthopedic doctors, epidemiologists, and rehabilitation physicians, who provided input regarding the study protocol. We registered our protocol in PROSPERO and reported our study following the Preferred Reporting Items for Systematic Reviews and Meta-analyses (PRISMA) and PRISMA-2020 guidelines and the extension statement for network meta-analysis (PRISMA-NMA)^[14,17]^. The presentation of our results adhered to the PRISMA, PRISMA-NMA, and Assessing the Methodological Quality of Systematic Reviews (AMSTAR)^[18]^ Guidelines.

### Literature review

In this NMA, we conducted a comprehensive literature search of Medline (PubMed), Embase, and the Cochrane Library to identify studies on surgical approaches for long bone defects published from 1 January 2000 to 12 January 2023. The complete search strategy for all databases is provided in Table S1 (http://links.lww.com/JS9/D840). Surgical treatments for bone defects include MT, BT, VBG, non-VBG (NVBG), and BTE. During the search period, we evaluated all relevant meta-analyses and systematic reviews to identify potentially suitable studies. Articles were imported into EndNote 20, and duplicates were removed. The review team independently screened the titles and abstracts of the studies to identify articles that met the inclusion criteria. The full texts of potentially suitable studies were evaluated based on the inclusion and exclusion criteria. Any disagreements were resolved by consulting a third reviewer^[19]^.

### Eligibility criteria

The study considered studies with (1) patients who had long bone defects, regardless of demographics or severity; (2) surgical treatments that included at least two of the following: MT, BT, VBG, NVBG, and BTE; (3) a randomized controlled or other controlled design; and (4) outcomes that included the bone defect healing rate or postoperative complication rate. We excluded (1) non-human studies, (2) uncontrolled studies (e.g., case reports, reviews, and conference submissions), (3) studies without extractable data, and (4) studies involving only one surgical method without comparison.

### Data extraction and risk of bias

All articles were screened by reviewers trained in systematic reviews and meta-analyses. The reviewers independently assessed the titles and abstracts of the articles retrieved from the search. Data extracted from eligible studies included publication year, journal, first author, study type, subject characteristics (sample size, average age, cause, location, and size of the bone defect), and intervention details (type, fracture healing rate, complications, and incidence of complications) (Table S2, http://links.lww.com/JS9/D840). Discrepancies were resolved through discussions until a consensus was reached. In cases of persistent disagreement, a third reviewer was consulted. Randomized clinical trials (RCTs) and observational studies were included. For observational studies, two independent investigators assessed the risk of bias using the Risk Of Bias In Nonrandomized Studies of Interventions (ROBINS-I) tool. For RCTs, two independent investigators assessed the risk of bias using the Risk of Bias 2 (RoB 2) tool^[20,21]^. Studies with a high risk of bias were excluded from the final analysis to ensure a fair estimation of the effect sizes.

### Statistical analysis

We considered the postoperative healing and complication rates of bone defects as outcome indicators. The primary outcome measure was the postoperative healing rate, with higher rates indicating better outcomes. The secondary outcome measure was the incidence of postoperative complications, with higher rates indicating poorer surgical safety. We calculated and reported the mean difference (MD) with corresponding 95% confidence intervals (CIs) using the Netmeta package in R, version 4.3.2, and Stata, version 17.0^[22]^. Clinical similarity, transitivity, and statistical consistency were assessed, and NMA estimates were calculated. League tables of relative treatment effect sizes were used to visualize network comparisons, and outcome measurements were determined after data collection. We conducted pairwise meta-analyses using the DerSimonian–Laird random-effects model to estimate the variance in heterogeneity and obtain direct evidence^[23]^. Network diagrams were created to establish the geometric relationships between treatments, with node size and line thickness representing participant numbers and direct comparison trials, respectively.

We compared the distribution of characteristics between the study groups to assess transitivity. The results were evaluated using consistency and inconsistency model methods. Inconsistency plots were used to assess the heterogeneity among studies in closed loops in the network meta-analysis. We applied the inconsistency factor (IF) to assess the heterogeneity among studies in each closed loop. If the 95% CI of the IF value was truncated to zero, the direction of the IF was not significant^[24]^. Node-splitting analysis was used to evaluate the local inconsistency between direct and indirect results^[25,26]^, with significant inconsistencies (*P* < 0.05) addressed through subgroup analysis.

The surface under the cumulative ranking curve (SUCRA) statistic was used to rank the effectiveness of different surgical interventions. The probability of each treatment being ranked at each level was estimated based on SUCRA values^[27]^. SUCRA identified the best surgical treatment for bone defects, minimizing the impact of small sample sizes. Funnel plots were evaluated to explore potential biases in small studies^[28]^.

## Results

### Results of the search and study characteristics

**Figure 1.**
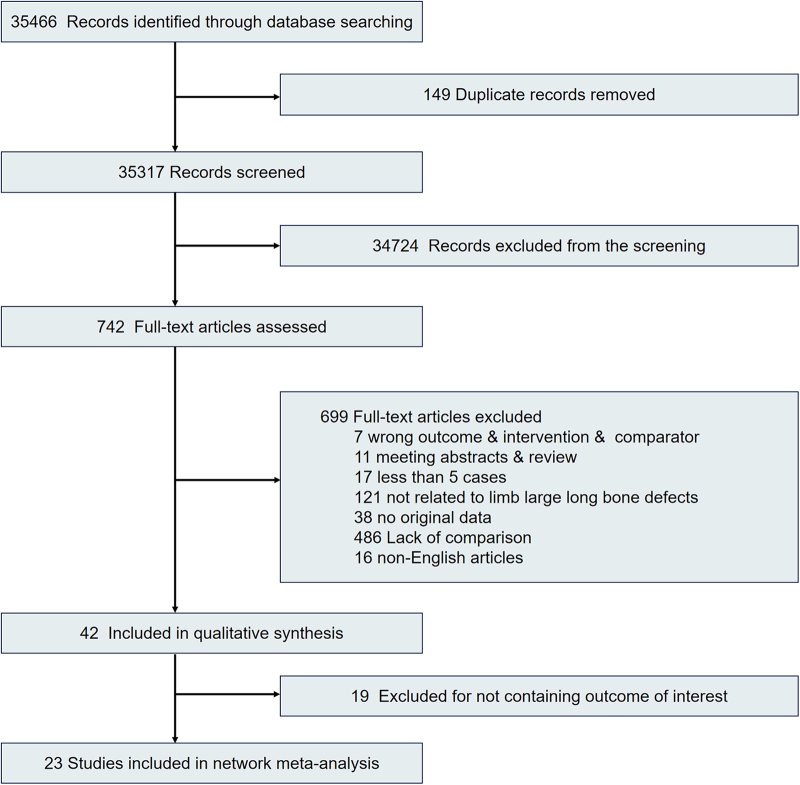
Flow diagram of the literature screening and selection processes.

A preliminary search identified 35 466 studies, of which 742 were eligible for full-text review. Forty-two articles initially met all the inclusion criteria; however, 19 were excluded because they did not contain the outcomes required for the study. Consequently, 23 articles were included in the analysis, comprising three RCTs^[29–31]^ and 20 observational studies^[32–51]^ (Fig. 1). Among them, 23 assessed the postoperative healing rates of patients with long bone defects in the limbs, and 18 evaluated the incidence of complications in patients with long bone defects. Of the included studies, eight evaluated MT, 13 assessed BT, 13 assessed VBG, 13 assessed NVBG, and 4 evaluated BTE. This analysis included 930 participants, with 763 and 130 cases of lower- and upper-limb reconstruction, respectively (tibia, 62.3%; femur, 17.9%; radius, 6.6%; humerus, 4.5%; ulna, 0.8%). Six articles reported a mixed series of lower- and upper-limb reconstructions; one article focused only on upper-limb reconstruction, and 16 articles focused only on lower-limb reconstruction. The mean bone defect was 8.1 cm (range 0.5–28 cm), and the age of the participants ranged from 7 to 62 years (IQR: 35 years). Patient characteristics are listed in Table S2 (http://links.lww.com/JS9/D840), without racial or ethnic information, as no relevant data on this regard were found in the included articles. A summary of the complications is reported in Table S3 (http://links.lww.com/JS9/D840). The main postoperative complications associated with the treatment of bone defects include nonunion, fractures, bone infections, and deformities.

### Risk of bias

**Figure 2.**
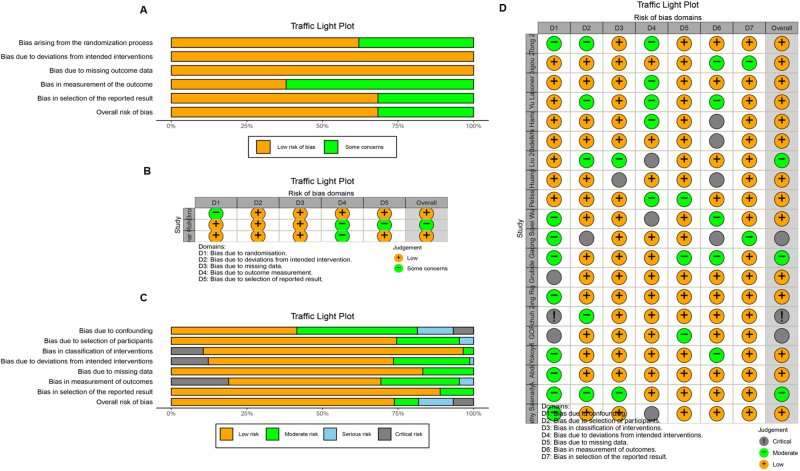
Assessment of risk of bias. (A) RoB 2 graph. (B) RoB 2 results for each article. (C) ROBINS-I risk of bias graph. (D) ROBINS-I risk of bias results for each article. Figure 2A-B RoB 2 bias assessment for RCTs. (C-D) ROBINS-I bias assessment for observational studies. Risk of bias summary: it is a summary table of review author’s judgments for each risk of bias item for each study. Risk of bias graph: it is a plot of the distribution of judgments across studies for each risk of bias item.

A risk-of-bias assessment was conducted, and the results are shown in Figure 2. Sixteen studies showed a low risk of bias, four showed a medium risk, and three indicated a high risk. In these studies, the risks of bias arose from (1) the randomization process, as it was unclear whether the allocation sequences were random and concealed^[5,52–54]^ and (2) deviation from the intended interventions, with uncertainty about whether participants or those delivering the interventions were aware of the assigned interventions during the trial^[5,55]^. In terms of outcome measurement, either the outcome measurements differed between the intervention groups^[52]^ or it was unclear whether the outcome assessors were aware of the interventions received by the study participants^[5,54,55]^.

### Network map

**Figure 3.**
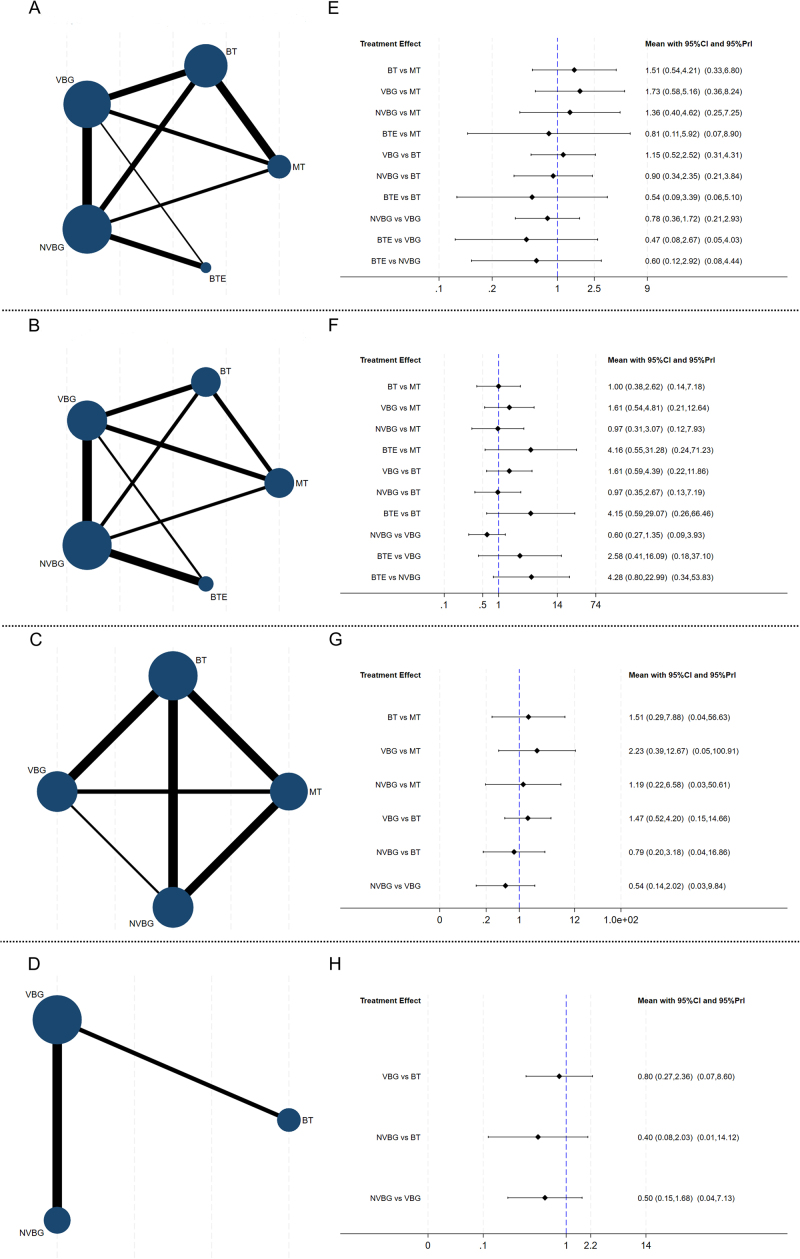
Network meta-analysis of surgical strategies for limb long bone defects. (A–D) Network evidence plot for bone defects. (E–F) Forest plot represents the direct and indirect comparison. From left to right are primary outcomes, secondary outcomes, and a subgroup analysis based on defect lengths of 4–8 cm and >8 cm. MT, masquelet technique; BT, bone transport; VBG, vascularized bone graft; NVBG, non-vascularized bone graft; BTE, bone tissue engineering.

**Figure 4.**
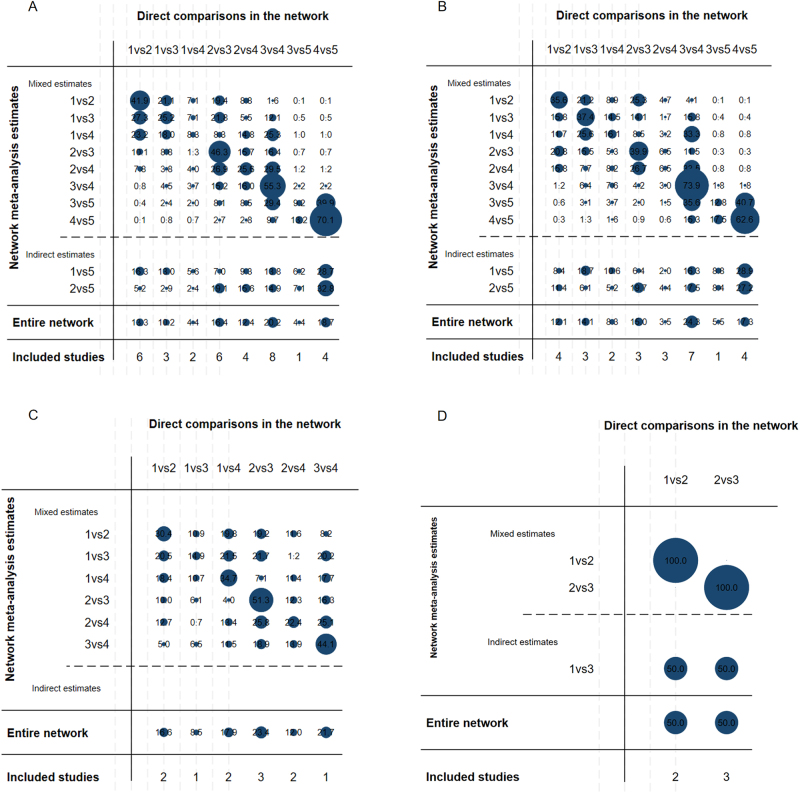
Contribution plot. (A) The primary outcomes. (B) The secondary outcomes. (C) A subgroup analysis based on defect lengths of 4–8 cm. (D) A subgroup analysis based on defect lengths of >8 cm. The contribution plot of the enrolled studies in this network meta-analysis. The size of each circle is proportional to the weight attached to each direct summary effect (horizontal axis) for the estimation of each network summary effects (vertical axis). The numbers re-express the weights as percentages.

A network nodal map was created for each outcome (Fig. 3A–D). The width of the lines represents the number of direct comparisons between treatments, and the size of the nodes represents the proportion of patients who received treatment. A contribution plot was used to evaluate the contribution of different direct comparisons to the NMA (Fig. 4). The size of each circle is proportional to the weight attached to each direct pooled effect (horizontal axis) used to estimate each network summary effect (vertical axis). In Figure S1 (http://links.lww.com/JS9/D840), the network forest plot in shows the MD and 95% CIs of all 22 individual direct-pair comparisons grouped in the five regimen pairwise meta-analyses.

By analyzing the data from these studies, we assessed the efficacy of five treatment regimens (Fig. 3E–H). Further details are provided in the league table (Fig. S2, http://links.lww.com/JS9/D840). We conducted pairwise comparisons of the efficacy of all treatment regimens for the primary and secondary outcomes. A comparison of the results is presented using MD with 95% CIs. No significant differences were observed in the efficacy of these treatments. Although we failed to reach statistical significance regarding the efficacy of various interventions, there was a tendency suggesting that VBG might be more effective in improving healing rates post-surgery for bone defects compared to the other four groups. This is evidenced by the following data: VBG vs. MT (MD, 1.73; 95% CI, 0.58–5.16), VBG vs. BT (MD, 1.15; 95% CI, 0.52–2.52), NVBG vs. VBG (MD, 0.78; 95% CI, 0.36–1.72), and BTE vs. VBG (MD, 0.47; 95% CI, 0.08–2.67) (Fig. 3E). Similarly, when considering the incidence of postoperative complications, although no statistically significant differences were found among the surgical comparisons, there was a tendency for BTE to have a higher complication rate compared to the other groups. This trend is supported by the data: BTE vs. MT (MD, 4.16; 95% CI, 0.55–31.28), BTE vs. BT (MD, 4.15; 95% CI, 0.59–29.07), BTE vs. VBG (MD, 2.58; 95% CI, 0.41–16.09), and BTE vs. NVBG (MD, 4.28; 95% CI, 0.80–22.99) (Fig. 3F).

### Ranking of treatments

**Figure 5.**
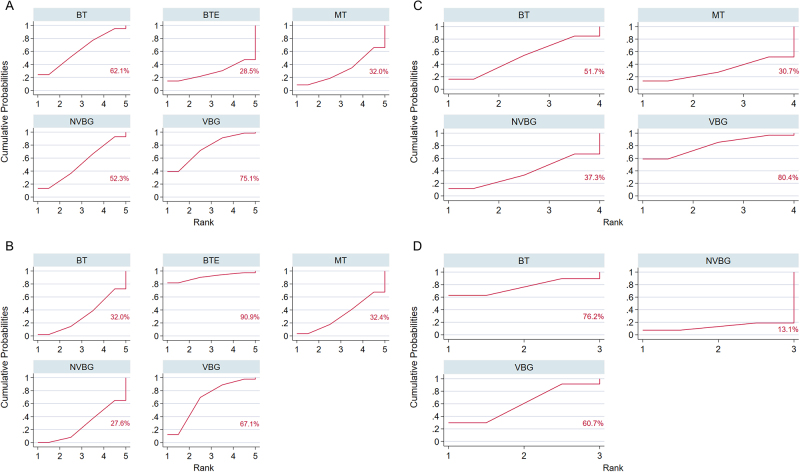
SUCRA values for treatments. (A) The primary outcomes. (B) The secondary outcomes. (C) A subgroup analysis based on defect lengths of 4–8 cm. (D) A subgroup analysis based on defect lengths of >8 cm. SUCRA (surface under the cumulative ranking curve) provides a numerical representation of the overall ranking of treatments. It assigns a value between 0% and 100% to each treatment, with higher SUCRA values indicating a greater likelihood of achieving the top rank or one of the top ranks. Conversely, lower SUCRA values suggest a greater likelihood of being ranked lower.

SUCRA values were calculated for each surgical intervention in terms of postoperative healing and complication rates of bone defects (Fig. 5). The results showed that regarding postoperative healing rates, VBG (SUCRA, 75.1%) ranked first, indicating the highest likelihood of being the most effective treatment, followed by BT (SUCRA, 62.1%), NVBG (SUCRA, 52.3%), MT (SUCRA, 32.0%), and BTE (SUCRA, 28.5%) (Fig. 5A). Concerning postoperative complication rates, BTE (SUCRA, 90.9%) ranked first, with the highest postoperative complication rate and thus the poorest treatment outcome, followed by VBG (SUCRA, 67.1%), MT (SUCRA, 32.4%), BT (SUCRA, 32.0%), and NVBG (SUCRA, 27.6%) (Fig. 5B).

### Publication bias and heterogeneity

Funnel plots were created to evaluate potential publication bias for the two outcome measures and the results of the subgroup analyses. The plots were symmetrical, indicating no significant publication bias (Fig. S3, http://links.lww.com/JS9/D840). In assessing heterogeneity among the closed-loop studies for the primary outcome measure, a statistical difference was observed in the loop MT-BT-VBG (IF, 2.20; 95% CI, 0.19–4.20) (Fig. S4, http://links.lww.com/JS9/D840), suggesting the possibility of statistical heterogeneity. Furthermore, local inconsistency tests using node-splitting methods showed significant differences between direct and indirect evidence for MT-BT (*P* < 0.05) and MT-VBG (*P* < 0.05) (Fig. S5, http://links.lww.com/JS9/D840), indicating inconsistency within the network. To identify the source of this inconsistency, we divided the primary outcome measures into two subgroups based on the size of the bone defect: 4–8 cm and >8 cm. Tests for inconsistency within these subgroups did not show significant differences between direct and indirect evidence (*P* > 0.05), suggesting that the bone defect size may be a key factor influencing the primary outcome measure.

### Subgroup analysis for the primary outcome

For the primary outcome measures, although the subgroup analysis did not show significant differences between the interventions, patients with bone defects of 4–8 cm appeared to benefit more from VBG than from MT and BT. The therapeutic effect of NVBG also seemed inferior to that of VBG (Fig. 3G). According to the SUCRA values, VBG (SUCRA, 80.4%) may have a better postoperative healing rate than other surgical interventions (Fig. 5C). BT was considered the best treatment method for patients with bone defects measuring >8 cm. The postoperative healing rates of VBG (MD, 0.80; 95% CI, 0.27–2.36) and NVBG (MD, 0.40; 95% CI, 0.08–2.03) were slightly worse than those of BT (Fig. 3H). According to the SUCRA values, BT (SUCRA, 76.2%) significantly outperformed the other interventions (Fig. 5D).

## Discussion

This NMA included the latest data from 20 observational studies and three RCTs. For the first time, these data aimed to evaluate the effectiveness (healing rate post-surgery for bone defects) and safety (incidence of postoperative complications) of five surgical interventions for treating long bone defects. We found no statistically significant differences in the effectiveness or safety of the five surgical interventions. Subsequently, we calculated SUCRA values for these interventions and conducted a ranking analysis. Our results indicated that VBG significantly improved the healing rate of bone defects postoperatively compared with other interventions. However, BTE is associated with a significantly higher risk of postoperative complications in patients with bone defects.

The size of bone defects significantly influences treatment strategies and disease outcomes^[5]^. In 1986, Schmitz *et al* introduced the concept of a “critical-sized bone defect” in an animal study^[56]^. Currently, the range of critical-size bone defects in humans is unclear. For instance, Keating suggested that bone defects >2 cm are unlikely to heal spontaneously^[57]^, while Schemitsch considered bone defects ≥2.5 cm to have a poor natural course^[58]^. Additionally, treating critically sized bone defects is often complex^[59,60]^, and large defects exceeding 4–5 cm pose significant challenges for orthopedic surgeons^[61]^.

In this study, heterogeneity analysis of the primary outcomes revealed significant heterogeneity. To explore the sources of this heterogeneity, we performed a subgroup analysis based on the size of bone defects. Tetsworth extended the fracture classification of the AO/Orthopedic Trauma Association to include a classification scheme for bone defects, which may help in selecting the most appropriate treatment strategy to optimize clinical outcomes^[62]^. Jorgensen clinically validated this classification system for post-traumatic bone defects and found substantial inter-observer agreement when assessing various bone defects following long open bone injuries, deeming the classification practical and applicable to trauma patients^[63]^. Therefore, we adopted this classification for our subgroup analysis and observed a significant reduction in heterogeneity, indicating that the size of the bone defect was a crucial factor affecting the primary outcomes. The subgroup results showed that VBG had a distinct advantage in treating segmental bone defects measuring 4–8 cm. BT is a more effective treatment method for bone defects >8 cm.

VBG can establish a complete blood supply through arterio-venous anastomosis post-transplantation, independent of the recipient bed’s blood supply. It bypasses the need for new bone callus formation and mineralization, requiring only the fusion of the graft’s ends, resulting in a lower bone resorption rate, faster healing, and shorter treatment duration. However, it also demands meticulous and complex surgical techniques, a high level of microsurgical expertise, and offers limited bone availability for extensive defects^[64,65]^. A review highlighted that the Ilizarov BT method demonstrates high union rates and lower complication rates when addressing critical-sized tibial defects, including those exceeding 8 cm. This study underscores the reliability and efficacy of BT in treating large bone defects^[45]^. It is important to note that while VBG and BT have certain advantages, the ranking of interventions based on absolute and cumulative effect probabilities in NMA may change with the addition of future evidence. Therefore, large-scale randomized controlled trials are still needed to further validate these findings.

Owing to the low heterogeneity of the secondary outcome indicator (incidence of postoperative complications), we did not perform a subgroup analysis. Based on SUCRA values, BTE demonstrated lower safety and efficacy. Possible reasons for this include (1) most research is still in the basic stage, making clinical application challenging in the short term; (2) limitations related to biosafety and ethical requirements; and (3) the high cost of new materials when introduced into clinical practice, restricting their widespread use^[60,66]^. However, BTE also possesses irreplaceable advantages, such as (1) adjustable physicochemical properties of the implants, (2) relatively abundant material sources, and (3) the ability to achieve personalized design of bone defect implants with macroscopic shape and internal microporous structure that can match the patient’s bone structure. The three-dimensional (3D)-printed ordered structural state of bredigite (BRT-O) scaffold exhibited excellent performance in promoting bone regeneration and immune regulation, particularly by enhancing M2 macrophage polarization, thus improving bone defect repair outcomes^[67]^. Shaowei *et al* designed a biocompatible hydrogel dynamically assembled from polyvinyl alcohol, borax, and tetraethyl orthosilicate (TEOS), which exhibited self-healing properties, low expansion rates, degradability, and good biocompatibility^[68]^. These studies suggest that BTE will have broader applications in the near future.

Moreover, it is worth noting that emerging novel techniques, including cell therapy, gene therapy, and microrobotic systems, offer transformative potential in the management of bone defects. Intraoperative tissue engineering, as described by Krasilnikova *et al*, enables the creation of tissue-engineered grafts directly in the operating room using minimally manipulated cells, providing a practical and accessible solution for advanced treatment^[69]^. Gene therapy, highlighted by Grol and Lee, utilizes viral and non-viral vectors to deliver therapeutic genes, promoting bone repair through growth factors and miRNAs, with successful clinical applications like *ex vivo* TGF-β1 gene therapy^[70]^. Additionally, microrobotic systems represent a cutting-edge approach for the musculoskeletal system, delivering stem cells or drugs with intelligent, precise, and minimally invasive targeting. As Cao *et al* noted, microrobots enhance local targeting efficiency through active motion, surpassing traditional biomaterial strategies^[71]^. Integrating these innovations with traditional surgical approaches could accelerate healing, improve graft integration, and address complex skeletal defects, paving the way for personalized and highly efficient treatment protocols.

### Study limitations

Our study has several limitations. First, while our initial search identified many studies, only a limited subset met the inclusion criteria, with just three being RCTs. This highlights the scarcity of high-quality, large-sample RCTs, which are essential for minimizing bias through randomization. Second, the inclusion of observational studies introduces inherent risks of residual confounding and bias due to the lack of random allocation. Although we used robust assessment tools (RoB 2 and ROBINS-I) and conducted sensitivity analyses, these limitations should be considered when interpreting the results. Third, some treatment comparisons relied on indirect evidence due to the limited availability of head-to-head studies, which may have introduced uncertainty. Fourth, the absence of standardized long-term follow-up data and functional outcome measures limits the ability to fully assess the impact of treatments on patient quality of life. Last, while SUCRA rankings provided valuable insights, they should be interpreted cautiously within the broader clinical and methodological context.

## Conclusions

Although no specific surgical intervention demonstrated significant superiority or inferiority in terms of healing rates and incidence of complications, this study offers valuable insights for clinicians in the decision-making process. Notably, VBG and BT exhibit potential advantages, possibly because of their positive roles in promoting bone healing and enhancing structural stability. Conversely, BTE may be associated with a higher risk of complications, warranting clinicians’ attention. However, these preliminary conclusions require further validation in high-quality, large-sample RCTs. Future research should focus on designing rigorous randomized trials that minimize bias and confounding factors, such as patient comorbidities and surgical expertise, to strengthen the evidence base.

## Data Availability

No additional unpublished data are available.

## Research registration unique identifying number (UIN)

We confirm that our study is registered in PROSPERO (CRD: 42023490571).
